# Genetic Evolution of *Mycobacterium bovis* Causing Tuberculosis in Livestock and Wildlife in France since 1978

**DOI:** 10.1371/journal.pone.0117103

**Published:** 2015-02-06

**Authors:** Amandine Hauer, Krystel De Cruz, Thierry Cochard, Sylvain Godreuil, Claudine Karoui, Sylvie Henault, Tabatha Bulach, Anne-Laure Bañuls, Franck Biet, María Laura Boschiroli

**Affiliations:** 1 Université Paris-Est, Laboratoire National de Référence de la Tuberculose, Unité de Zoonoses Bactériennes, Laboratoire de Santé Animale, ANSES, Maisons-Alfort Cedex, France; 2 INRA, UMR1282, Infectiologie et Santé Publique (ISP-311), Nouzilly, France; 3 INSERM U1058 Infection par le VIH et par agents à tropisme cutanéo-muqueux: de la pathogenèse à la prévention, Montpellier, France; 4 Centre Hospitalier Régional Universitaire de Montpellier, Hôpital Arnaud de Villeneuve, Département de Bactériologie-Virologie, Montpellier, France; 5 MIVEGEC, UMR IRD 224-CNRS 5290-Universités Montpellier 1 et 2, Centre IRD, Montpellier, France; Institut national de la santé et de la recherche médicale—Institut Cochin, FRANCE

## Abstract

To study the dynamics of bovine tuberculosis (bTB) in France, 4,654 *M. bovis* strains isolated mainly from livestock and wildlife since 1978 were characterized by spoligotyping and MLVA based on MIRU-VNTR. In our study spoligotyping allowed the discrimination of 176 types although 3 spoligotypes are predominant and account for more than half of the total strain population: SB0120 (26%), SB0134 (11%) and SB0121 (6%). In addition, 11% of the isolates, principally from Southern France, showing close spoligotypes and MIRU-VNTR types have been gathered in a family designated as the “F4-family”. MLVA typing allowed extensive discrimination, particularly for strains with predominant spoligotypes, with a total of 498 genotypes, several of which were highly regionalized. The similarity of the strains’ genetic relationships based on spoligotyping and MIRU-VNTR markers supports the co-existence of different clonal populations within the French *M. bovis* population. A genetic evolution of the strains was observed both geographically and in time. Indeed, as a result of the reduction of bTB due to the national control campaigns, a large reduction of the strains’ genetic variability took place in the last ten years. However, in the regions were bTB is highly prevalent at present, cases in both livestock and in wildlife are due to the spread of unique local genotype profiles. Our results show that the highly discriminating genotyping tools used in this study for molecular studies of bTB are useful for addressing pending questions, which would lead to a better insight into the epidemiology of the disease, and for finding proper solutions for its sustainable control in France.

## Introduction

Bovine tuberculosis (bTB) is a worldwide distributed chronic disease which affects significantly both cattle productivity and trading of live animals or animal products [1–3]. bTB main agent is *Mycobacterium bovis*, a very successful pathogen that belongs to the *Mycobacterium tuberculosis* complex (MTBC) [4]. Cattle constitute the main hosts reservoir for this bacterium but other domestic animals and wildlife species can be infected [5]. Albeit recent findings showing that compared to *M*. *tuberculosis*, mutations on the PhoPR regulation system in the MTBC lead to a decreased affinity of *M*. *bovis* to humans, [6,7], this species remains, nevertheless, a significant human pathogen of particular importance in bTB endemic regions in the world [3,8,9]. During the 20^th^ century, control programs based on cattle “test and slaughter” were highly successful in controlling bTB in most European Union (EU) countries or eradicating it from some of them [10] and, together with introduction of milk pasteurization, they lead to the strong decrease of human tuberculosis cases due to *M*. *bovis* [11]. However, in the last decades, bTB has re-emerged in the EU, mainly in countries where *M*. *bovis* wildlife serves as reservoir of the pathogen thus hampering its control in livestock [12–14].

In France, bTB was endemic until the 50s with a herd prevalence rate of 25% [15]. Thanks to a generalized control program set up in the 1960s, bTB herd prevalence declined steadily until reaching 0.09% in 1998. In 2000, France was recognized officially free of bTB by the EU [16]. However, in the last 10 years the evolution of the monitoring methods and surveillance pressure both in livestock and wildlife lead to discover an increasing number of cases in certain French areas in both animal populations.

To examine the evolution of *M*. *bovis* infection in space and time in the country, it is essential to study genetic differentiation of isolates and their phylogenetic relationships together with cattle movement and wildlife ecology. Genotyping method based on slowly mutating markers, such as spoligotyping, studying the deletion of “spacer” sequences in the MTBC Direct Repeat (DR) region [17], is suitable for inferring the evolutionary history of isolates. In another way, genotyping based on more rapidly evolving markers, such as the study of the number of variation of tandem repeats (MIRU-VNTR) in particular *loci*, is well suited to tracing transmission and investigating outbreaks’ origins [18–20].

In a previous study, the diversity of the totality of *M*. *bovis* strains isolated in France between 1979 and 2000 was established by spoligotyping [21]. This study included 1,349 isolates and revealed a wide diversity of genotypes, with BCG-like (actual SB0120) spoligotype described as predominant. The study also highlighted that some spoligotypes were closely associated with restricted geographical areas.

In the present study, we report the results of the French *M*. *bovis* strains diversity isolated from 1978 to 2013 by using a combination of spoligotyping and MIRU-VNTR typing. This study included livestock strains selected at a herd-based level and most importantly wildlife isolates discovered since 2000, in order to better explore the genetic diversity of *M*. *bovis*.

The final aim was to use genotyping analyses to investigate the structure of the current *M*. *bovis* population based on the retrospective analysis of bTB in cattle and wildlife thus allowing investigation of detailed epidemiological issues and generating appropriate surveillance questions to explain the apparent reemergence of the disease in France.

## Materials and Methods

### Ethical statement

bTB is a notifiable disease for which there are control and surveillance campaigns in France. Official methods for diagnosis of this disease are culture, PCR and histopathology. Therefore, all the datasets included in this study are issued from animals analyzes within an official context. No purpose killing of animals was performed for this study. All datasets were in complete agreement with national and European regulations. No ethical approval was necessary.

### Survey

The strains analyzed in this study were routinely collected by the National Reference Laboratory (NLR) for Bovine Tuberculosis on behalf of the French Agriculture Ministry in the framework of the livestock eradication program and the wildlife surveillance program. For other animal species (zoo species, domestic animals), isolation was performed upon discovery of bTB-like lesions at post-mortem examination. For livestock, infected animals were detected by *M*. *bovis* bacterial culture after (i) being slaughtered for diagnostic purposes follows a previous positive skin or IFN-Ɣ testing; (ii) having presented macroscopic bTB-like lesions at routine abattoir inspection. Wildlife surveillance programs had been conducted in France in regions where cattle bTB was important [22–24] and currently under the framework of the Sylvatub plan recently launched in 2011 (NOTE DE SERVICE DGAL/SDSPA/N2011–8214, Date: 20 September 2011).

For cattle or other farm animal breakdowns, a herd-based criterium was used for strain choice: at least 1 strain/outbreak was genotyped by both genotyping methods. If there is more than one spoligotype profile in the same herd during one year, two strains of each spoligotype profiles were fully genotyped (MLVA-typed and spoligotyped). The totality of wildlife, zoo or other domestic animal isolates were fully genotyped by both genotyping methods (spoligotyping + MIRU-VNTR). Thus, 88% of the strains were isolated from cattle, 9% from wild animals (badgers, red-deer, wild boar), 3% from other farm animals and the rest from zoo or domestic animals (cats, dogs).

Prevalence and incidence data were obtained from the French Ministry of Agriculture, Food and Forestry (MAFF).

### *Mycobacterium bovis* isolation

Bacterial culture was performed according to the protocol established by the French NRL (NF U 47–104) for isolation of *M*. *bovis*. After decontamination, the supernatant was seeded on two different media: Löwenstein-Jensen and Coletsos [25]. All seeded media were incubated at 37°C +/- 3°C for 3 months and examined every 2 weeks. The isolated *Mycobacterium tuberculosis* bacterial complex (MTBC) colonies were confirmed by DNA amplification as described by Hénault *et al*. [26] and *M*. *bovis* was confirmed by spoligotyping (see below).

### Genotyping methods

***Spoligotyping***

Spoligotyping, as described by Kamerbeek *et al*. [27], was performed in 4,020 of the 4,654 *M*. *bovis* identified strains. The spacer sequences contained in the DR locus were detected by hybridization onto spoligotyping membranes (Isogen Bioscience BV, Maarssen, The Netherlands) until 2010. Since 2011 spoligotyping has been performed by Luminex as described by Zhang *et al*. [28], by hybridization onto fluorescent beads presented to laser detection. The presence or absence of the 43 spacers contained in the DR locus was represented in a binary code of 43 entries. Spoligotypes were named according to an agreed international convention (www.mbovis.org).

### MLVA typing using MIRU and VNTR markers

Mycobacterial interspersed repetitive units- Variable numbers tandem repeats (MIRU-VNTR) profile identification [29,30] was performed by Genoscreen, Lille, France, using PCR amplification targeting genetic loci with mycobacterial MIRU-VNTR. Analysis was based on 8 *loci* (2165, 2461, 0577, 580, 2163a, 2163b, 4052 and 3232) chosen in the framework of a European consortium and on their degree of polymorphism and their ability to discriminate local strains [31]. In this strudy, 2,332 *M*. *bovis* isolates were analyzed by this method.

### Genotype analysis

The Bionumerics software 7.0 (Applied Maths, St-Martin-Latem, Belgium) was used to analyze MIRU-VNTR patterns and spoligotype patterns. They were investigated by drawing minimum spanning trees (MST) and dendrogrammes (categorical value distance represented by UPGMA clustering method for MIRU-VNTR or spoligotype, Multi-state categorical correlation represented by UPGMA clustering method for MIRU-VNTR plus spoligotype).

The discriminatory index power (DI) described by Hunter and Gaston was used as a numerical index for the discriminatory power of each typing method [32]. The DI was calculated using the formula DI = 1− (1÷N (N−1)Σ^*s*^_*j* = 1_ n*j*(n*j*−1)) where *N* is the total number of strains in the typing scheme, *s* is the total number of distinct patterns discriminated by each typing method and strategy, and *nj* is the number of strains belonging to the *j*th pattern.

### Geographical analysis

Maps were drawn using Quantum GIS 2.0.1 Dufour program. The French territory was divided in “departments” (administrative regions). Percentages were calculated by the proportion of strains isolated by “department” at a given period of time compared to the total number of strains isolated in the whole territory in the same period.

## Results

### Demography of *M*. *bovis*

**Fig 1:**
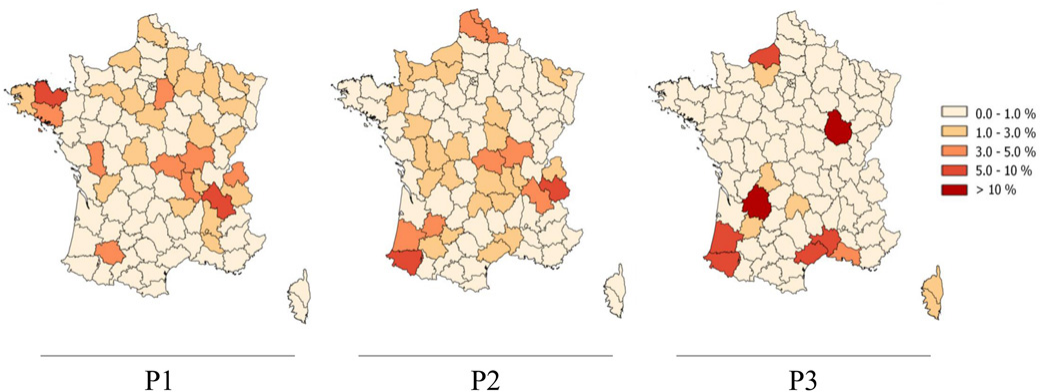
Geographical distribution of endemic outbreaks of bovine tuberculosis in France. Color intensity reflects the percentage of *M*. *bovis* strains isolated in each “department” compared to the totality of *M*. *bovis* isolated in the whole territory for the given period of time. The first map represents the 377 *M*. *bovis* strains isolated in France from 1978 to 1990. The second map represents the 1,176 *M*. *bovis* strains isolated in France from 1991 to 2000. The third map represents the 3,101 *M*. *bovis* isolated in France from 2001 to 2013.

From the late 70s and the establishment of a reinforced bTB control policy, the incidence rates of the disease decreased steadily until 2004. In 2000 France finally obtained the bTB free status from the EU. This 35-years retrospective study was divided into 3 periods: period 1 (1978–1990); period 2 (1991–2000) and period 3 (2001–2013). Over periods 1 and 2, the geographical distribution of *M*. *bovis* strains in France was quite homogeneous all over the whole territory (Fig. 1). In the third period, the distribution changed, becoming more localized (Fig. 1). Indeed, since the 2000s, the infection has concentrated mainly in 2 regions, Côte d’Or (Middle-East) and Dordogne (South-West) that account for almost 40% of cases detected in the last 10 years. Infection has persisted in Camargue (South), Normandy (Middle-North) and the South-West (Fig. 1).

**Fig 2:**
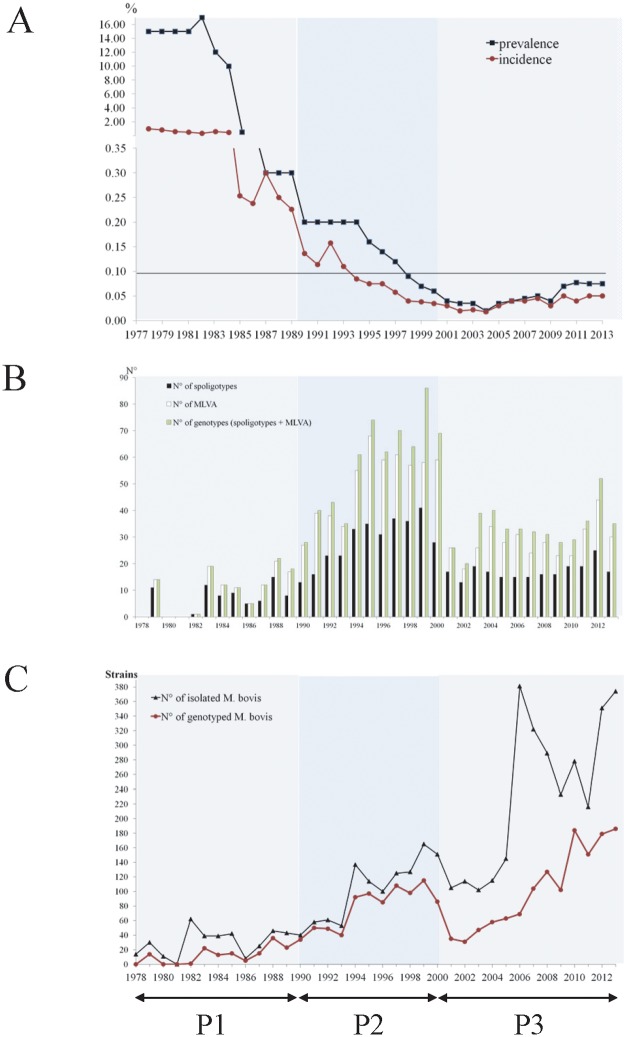
Evolution of the prevalence and the incidence rate of bTB (A), of the different strain profiles number (B) and yearly evolution of the strain collection (C) of M. bovis in France from 1978 to 2013. A: On the abscissa are represented the years, on the ordinate, the percentage of incidence (◼) and prevalence (●). Black line cutting the ordinate axe at 0.1% represents the limit under which an EU country is considered free of bTB. Below the graph, three lines (P1; P2; P3) represent the three periods studied. B: In black bars, spoligotypes, in white bars, MLVA profiles, in green bars, the association of both methods. C: Yearly evolution of the number of strains collected (Black) and spoligotype-MLVA identification (Pink) of the 4,654 *M*. *bovis* strains received in the National Reference laboratory between 1978 and 2013. For both graphs, lines represent the three studied periods (P1; P2; P3).

Over the period 1978–2013, 2,332 strains of *M*. *bovis* were genotyped by spoligotyping and MLVA, leading to the identification of 706 different genotypic profiles (Fig. 2B). During the first period, 132 different genotypes were characterized from 178 strains. During the second period, 418 genotypes were identified from 820 strains. During the third period, 256 genotypes were determined on 1,334 strains. During the last period, the number of isolates increased significantly (Fig. 2C). This rise in strain recruitment was the consequence of the larger number of animals investigated per herd upon bTB suspicion but also due to the generalized surveys in wildlife set up since 2001. Despite this increase in the number of strains, prevalence/incidence rates of the disease never went beyond the limit of 0.1% and was the least important in the 3 periods (Fig. 2A) with a consequent bacterial genotypic diversity decrease over time (Fig. 2B).

### Genotypic evolution of *M*. *bovis* population in space and time

**Fig 3:**
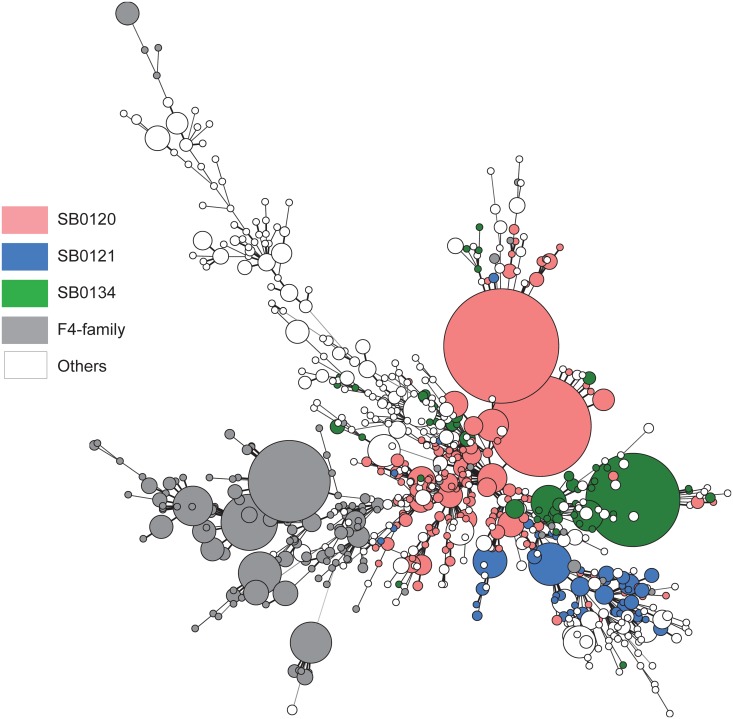
Minimum spanning tree (MST) of the 2,332 *Mycobacterium bovis* isolates between 1978 and 2013. 706 genotypes were obtained by combination of spoligotyping and VNTR typing. The colored nodes show the principal families of *M*. *bovis*, SB0120 (Pink), SB0121 (Blue), SB0134 (Green) and “F4-family” (Grey). Distances between nodes represent distances between profiles.

Spoligotyping analyses were performed on 4,020 *M*. *bovis* strains collected in the NLR since 1978 (Fig. 3 and S1 Dataset); 377 between 1978 and 1990, 1,176 between 1991 and 2000 and 3,101 between 2001 and 2013. This method allowed the discrimination of 176 spoligotypes (including 15 new profiles compared to the 2000s study [21]), but 3 predominant types, SB0120, SB0134 and SB0121, covered almost half of the isolates, representing respectively 26%, 11% and 6% of the total strain population. In addition, 11% of the spoligotyped isolates, principally originated from Southern France, with common features in the DR locus and MIRU-VNTR types, have been gathered in a group designated as the “F4-family” (Fig. 3).

### Spoligotype SB0120 analysis

SB0120 is the most common and widespread spoligotype in France. Of the 1,230 SB0120 strains identified between 1978 and 2013, 778 were typed by MLVA, showing 154 different MIRU-VNTR profiles corresponding to a DI of 0.858 (Table 1 and S1A Fig. and S1 Dataset).

From 1978 to 1990, 117 SB0120 strains were identified (31% of the total population), 40 MIRU-VNTR genotypes were identified on 64 strains (DI: 0.961), located homogeneously in France. Between 1991 and 2000, SB0120 strains account for 29% of the total *M*. *bovis* population, principally located in the North of France and in Savoy (Middle-East). MLVA typing was performed on 185 strains and discriminated 91 MIRU-VNTR genotypes with a DI of 0.986, the highest for this spoligotype, reflecting the important diversity of this group during the second period. In the last period, 771 SB0120 strains (25%) were identified, of which 529 resolved into 53 different MIRU-VNTR genotypes (DI: 0.704). They were mostly found in Dordogne (South-West) and Côte d’Or (Middle-East).

**Table 1:** Detailed spoligotypes: number of strains, discrimination by MLVA MIRU-VNTR typing.

Spoligotype	Periods over 1978–2013	N° of strains identified (% of total 4,654 *M*. *bovis* identified)	N° of genotype MIRU-VNTR (DI)
	*1978–2013*	778 (26.43)	154 (0.858)
	P1 1978–1990	64 (31.05)	40 (0.961)
**SB0120**	P2 1991–2000	185 (29.08)	91 (0.986)
	P3 2001–2013	529 (24.86)	53 (0.704)
	*1978–2013*	270 (11.45)	53 (0.677)
	P1 1978–1990	21 (24.93)	17 (0.981)
**SB0134**	P2 1991–2000	40 (7.8)	23 (0.945)
	P3 2001–2013	209 (11.19)	20 (0.47)
	*1978–2013*	154 (6.49)	50 (0.931)
	P1 1978–1990	10 (14.32)	6 (0.844)
**SB0121**	P2 1991–2000	81 (11.09)	31 (0.942)
	P3 2001–2013	63 (3.48)	21 (0.878)

Among the 154 MIRU-VNTR genotypes identified between 1978 and 2013, two which account for more than 52% of the totality stand out (Table 2 and S1A Fig.): profile-5 3 5 3 9 4 5 6, mainly present in Dordogne and neighboring regions (South-west), and profile-5 5 4 3 11 4 5 6, specifically located in Côte d’Or (Center-east) albeit being widely represented in the last period.

**Table 2:** Spatial distribution of genotypes of *M. bovis* isolated from multiple hosts.

Region of isolates	Location	Spoligotype	Genotype	Multi-host origin of isolates
			MIRU-VNTR	
Ardennes	North-East	SB0120	5 3 5 6 11 4 6 8	Cattle, Badger
Ariège	Middle-South-West	SB0134	6 5 5 3 6 4 5 6	Cattle, Wild boar
Côte d’Or	Middle-East	SB0120	5 5 4 3 11 4 5 6	Cattle, Badger, Wild boar
		SB0134	6 4 5 3 6 4 3 6	Cattle, Badger, Deer,Wild boar
Corsica Island	Mediterranean Isle	SB0840	7 5 5 3 8 2 5s 4	Cattle, Wild boar
		SB0120	4 5 5 3 11 4 5 7	Cattle,Wild boar
Dordogne & Charente	Middle-West	SB0120	5 3 5 3 9 4 5 6	Cattle, Badger, Deer, Wild boar
Dordogne		SB0999	6 4 5 2 8 2 4 7	Cattle, Badger, Wild boar
Normandy	Middle-North	SB0134	7 4 5 3 10 4 5 10	Cattle, Wild boar
Atlantic Pyrenees	South-West	SB0821	6 5 5 3 11 2 5s 8	Cattle, Badger, Wild boar
Landes	South-West	SB0821	6 5 5 3 11 2 5s 8	Cattle, Badger
Lot et Garonne	South-Middle-West	SB0823	6 5 5 3 11 2 5s 6	Cattle, Badger

These most common profiles are shared by livestock and wildlife (discussed below). Over time, some MIRU-VNTR genotypes appeared, some disappeared, and some were present during the 3 periods (S1B Fig.). The most important examples are the highly localized MIRU-VNTR types of the Alpine region (South-East), which were actively present during the first two periods but then decreased drastically after the 2000s just as bTB decreased in general in this region, which used to be highly prevalent to the disease beforehand (Fig. 1).

SB0120 was widespread all over the country but, in the last 10 years, specific MLVA-profiles seem to spread in particular regions as depicted in S1B Fig.

### Spoligotype SB0134 analysis

SB0134 is the second most common spoligotype in France. Of the 533 SB0120 strains identified between 1978 and 2013, 270 were typed by MLVA, showing 53 different MIRU-VNTR profiles (Table 1 and S2A Fig. and S1 Dataset). With a DI at 0.677, this spoligotype is the less diverse in MLVA-types when compared to the other two main groups (S2 Fig. and Table 1).

From 1978 to 1990, 94 SB0134 strains were identified (25% of the total population), 17 MIRU-VNTR genotypes were identified on 21 strains genotyped (DI: 0.981, the highest for this spoligotype), widespread over France. Between 1991 and 2000, SB0134 strains accounted for 8% (92 SB0134 identified) of the total *M*. *bovis* population, spread throughout the French territory. MLVA typing performed on 40 strains and discriminated 23 MIRU-VNTR genotypes with a DI of 0.945. In the last period, 347 SB0134 strains (11%) were identified, of which 209 resolved into 20 different MIRU-VNTR genotypes (DI: 0.470). They were mostly found in Côte d’Or (Middle-East) and Normandy (Middle-North).

Among the 53 MIRU-VNTR genotypes identified between 1978 and 2013, three stand out (Table 2 and S2A Fig.). Profile-6 4 5 3 6 4 3 6 (56% of all SB0134), mainly found in Côte d’Or (Middle-East), although also present in several other regions, is found in cattle as well as in wildlife species. The MLVA-profile-7 4 5 3 10 4 5 10 is the genotype of the strains isolated in the Brotonne Forest and its neighboring zones, in Normandy (Middle-North). Strains with this genotype infected wild boar, deer and cattle. Profile-6 5 5 3 6 4 5 6 corresponds to strains causing many cases of bTB in the regions bordering the departments of Ariège and Haute Garonne (Middle-South-West). As for the two other profiles, it infects wildlife and bovine (Table 2 and S2B Fig.).

### Spoligotype SB0121 analysis

SB0121 is the third most common spoligotype in France. Of the 302 SB0121 strains identified between 1978 and 2013, 154 were typed by MLVA, showing 50 different MIRU-VNTR profiles (Table 1 and S3A Fig. and S1 Dataset).

From 1978 to 1990, 54 SB0121 strains were identified (14% of the total *M*. *bovis* population), 6 MIRU-VNTR genotypes were identified on 10 strains (DI: 0.844), principally located in Brittany (North-West). Between 1991 and 2000, SB0121 strains account for 11% of the total *M*. *bovis* population, representing 140 strains identified as SB0121, principally located in the North of France. MLVA typing was performed on 81 strains and discriminated 31 MIRU-VNTR genotypes with a DI of 0.942, the highest for this spoligotype, reflecting the high diversity of this group during the second period. In the last period, 108 SB0121 strains (3.5%) were identified, of which 63 resolved into 21 different MIRU-VNTR genotypes (DI: 0.878). They were mostly found in Western France.

Among the 50 MIRU-VNTR genotypes identified between 1978 and 2013, the most common MIRU-VNTR genotype (21%), profile-6 4 5 3 11 2 5 7, is isolated since 1990 (S3A Fig.). This genotype was identified among cattle and farm deer, mostly in Brittany (North-West) and Normandy (Middle-North). Since 2011 it was not found any more in its original regions, but it was spread all over the territory.

The second SB0121 MIRU-VNTR type, profile-5 2 5 3 8 2 5 6, accounts for 13% of all genotyped SB0121 strains and has been isolated specifically in cattle. It was present in the Atlantic-Pyrenees (South-West) in 1996 and became very prevalent in the last few years, starting spreading to the Midi-Pyrénées (South) in 2013 (S3B Fig.).

SB0121 was not specifically isolated in a particular region, and unlike the two other spoligotypes described previously, it does not seem to fix and spread locally.

### Study of a special family of strains: the “F4–family”

**Fig 4:**
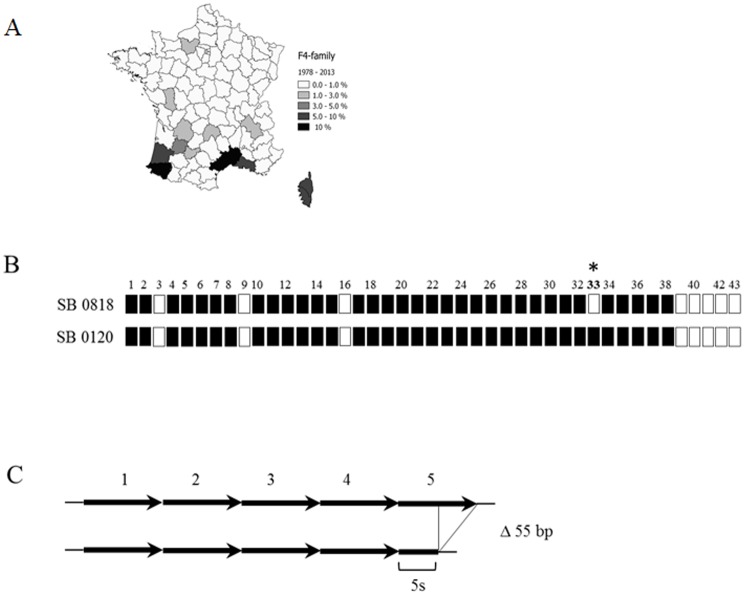
Definition of “F4-family”. (A) Geographical location: in the map, shades of grey indicate the percentage of isolates of the“F4 family” spoligotypes from the total *M*. *bovis* population, showing a principal localization in the South of France. (B) Spoligotype particularity: the chosen spoligotype, SB0818, the likely common ancestor of this family, is characterized by classic *M*. *bovis* missing spacers 3, 9, 16, and 39 to 43 plus the absence of the spacer 33, specific to the “F4-family”. (C) VNTR characteristics: “F4-family” possesses a truncated repetition (30 bp instead of 56 bp) in locus 4052, which is counted for the allele definition. In the example, other strains possess five repetitions but in the case of “F4 family”, the fifth repetition is truncated and is represented as a “5s”.

This group of strains is defined both by the absence of spacer 33 in the DR region and the presence of a truncated repeat (indicated by an "s") in the MIRU-VNTR locus 4052 (Fig. 4 and S1 Dataset). Strains of this family are located mainly in the south of France. Between 1978 and 2013, 530 strains of this family were identified, representing 23% of the genotyped studied *M*. *bovis* collection. This family includes 25 spoligotypes profiles, 106 MIRU-VNTR profiles summing up 137 different genotypes. Members of this group have already been described as a cluster in the South of France by Haddad *et al*. [21]. On the MST combining spoligotyping plus MIRU-VNTR typing (S4A Fig.), it is noteworthy that, except for the “reference” “F4-family” spoligotype, i.e. SB0818 (previously named F4 [21]), which is quite varied in MLVA diversity, MIRU-VNTR types of the other “F4-family” spoligotypes are rather restricted. Thus, for practical purposes, the evolution of the “F4–family” in time was thus undertaken only with spoligotype data.

From 1978 to 1990, 12 strains of the “F4–family” were found, with 7 different spoligotypes, which accounted for 3% of the total *M*. *bovis* strains population isolated in France over this period. During the second period, 150 strains with 21 different spoligotypes of this group were found, which accounted for 13% of the total population of strains isolated during this period. Between 2001 and 2013, 368 strains, i.e. 12% of the total population in this period, were identified and characterized by 13 different spoligotypes.

Many spoligotypes of this family are associated with a particular region where they were found both in livestock and wildlife (S4B Fig.). Spoligotype SB0840 is exclusive of the Corsica Island (Mediterranean island). Spoligotype SB0821 is endemic in Landes and the Atlantic Pyrenees regions (South-West), while spoligotypes SB0832 and SB0826 are also present in the latter; SB0818 is found in several localizations in Middle South. Spoligotype SB0823 is specifically found in Lot et Garonne (Middle-South-West). SB0825 and SB0827 are the main strains of the Camargue region (Middle-South).

Strains of the “F4 family” are poorly represented in the collection of strains of the first period studied albeit they could be more accurately identified in the last 20 years, always with a preferential localization in Southern France. However, most of the “F4-family” spoligotypes found at present had already been identified in the 1978 to 1990 period (S4 Fig.). Once more, and as for the main spoligotypes described previously, variations in the spoligotype diversity of the strains were observed between periods 2 and 3, with the same trend of genotype reduction and mainly disappearance of certain genotypes in time (S4 Fig.).

### *Mycobacterium bovis* in multi-host systems

Since 2000, with the discovery of the first outbreak of bovine tuberculosis in wildlife in France [23], many studies have been undertaken to investigate the presence of the bacterium in wildlife species, especially in regions with endemic tuberculosis in cattle. All these studies leading to the identification of numerous TB cases in wildlife which have largely contributed to the enlargement of the NRL’s strain collection (Fig. 2C). The types of *M*. *bovis* strains shared by different animal species are summarized in Table 2.

It is noteworthy that in the regions where wildlife cases are found, the local spread of specific genotypes is observed. This applies to the Brotonne Forest (Normandy), region where the first French case of bTB in wildlife was detected, the profile SB0134–7 4 5 3 10 4 5 10 is actually isolated from deer but also from cattle. SB0134–6 5 5 3 6 4 5 6 was isolated in Ariège-Haute Garonne in cattle and wild boar. In Côte d’Or, two major strains circulate among cattle, wild boar, deer and badgers: SB0120–5 5 4 3 11 4 5 6 profiles and SB0134–6 4 5 3 6 4 3 6 profile. In Dordogne and Charente the main *M*. *bovis* strain isolated from cattle, badgers and wild boar is SB0120–5 3 5 3 9 4 5 6. In members of the “F4-family” SB0821 with a MIRU-VNTR profile-6 5 5 3 11 2 5s 8 is shared by badgers, wild boar and cattle in the Atlantic Pyrenees and Landes or with SB0823–6 5 5 3 11 2 5s 6, isolated from badgers and cattle in Lot et Garonne. In Corsica, the two main types SB0120–4 4 5 3 11 4 5 7 and SB0840–7 5 5 3 8 2 5s 4 are shared both by cattle and wild boar.

Thus the uniformity of genetic profiles found in both wildlife and livestock within a region supports the hypothesis of inter-species transmission with a lack of host-specificity barrier for *M*. *bovis* transmission independently of the genotype.

## Discussion

To study the dynamics of French bTB, spoligotyping and MIRU-VNTR typing were used to genotype a wide collection of *M*. *bovis* strains isolated from livestock, wildlife or other animal species, from 1978 to 2013. In this way, more than 700 genotypes of *M*. *bovis* were identified. As described in this study and a previous one [21], a wide variety of *M*. *bovis* genotypes are inherent to specific geographical sites. Besides, strong fluctuations in the genetic variability of *M*. *bovis* over time and depending on different geographic areas were found.

Although our collection is well represented as far as time, space and animal hosts are concerned, the strain set of the first period (1978–1990) is quite restricted even if bTB in cattle was maximal at that time. Indeed, bacteriology became systematic upon bTB suspicion but only in the 90’s. Before that, bTB declaration was largely performed upon positive skin test results or the discovery of bTB like lesions at the abattoir. Thus, a retrospective genotype comparison over the whole studied period is partial.

Our study highlighted a sharp reduction in the genetic variability of strains in the past decade. This phenomenon may be related to the effectiveness of the bTB control program responsible for the disappearance of the disease in several French regions. Indeed, a better control of the disease produces classically a population bottleneck of the pathogen, leading to a decrease of the genetic diversity. In this process, only a limited subset of the pathogen’s genotypes present before implementation of control measures will be detected after applying them. Indeed, most of the genotypes associated at present to epidemic outbreaks were firstly identified during the second period. However, it is not possible to determine if such a bottleneck of predominant strains took place or if the strains were prone to spread anyhow as a result of particular virulence or transmissibility traits. The reduction of genotype diversity can also be explained by changes in herd practices introduced in the last 20 years, mainly the professionalization of breeders’ activity. Giving up rearing of certain ancient local breeds, which might have been host of specific *M*. *bovis* strains, for other more productive breeds is one of these changes. Strikingly, in the last decade, the majority of cases are due to a restricted number of *M*. *bovis* genotypes. These particular genotypes seem to proliferate in multi-host component systems including livestock and wildlife, at a quite restricted local level. Besides, the analysis of the bTB distribution and evolution in France during the last decade shown that current outbreaks in geographical locations are due to local spread of bacteria infected beef herds more frequently than dairy herds.

Risk factors related to suckler farming greatly developed in France in the last decades-taking over milk farming as in other EU countries, where bovine tuberculosis seems to focus at the present time, can partly explain this demographic variation of *M*. *bovis* population in the studied period. Indeed, suckling farms present strong bTB risk factors, such as operating farm over several premises, large land coverage, long stay in pastures that favors neighboring risk and environmental contamination by wildlife contact [33]. Difficulties are associated to the reduced man surveillance power of big herds in animals which are more difficult to contain than milk cattle, thus rendering veterinary handling more delicate [34]. The predominance of a particular genotype may reflect a better strain adaptation to the environment and/or to the host[35]. In parallel to the reduction of genetic variability, there is a decrease in the prevalence of the disease in France albeit the greater number of isolates that had been collected over time as a consequence of improvements in TB monitoring.

In this study, the 3 most common spoligotypes of *M*. *bovis* distributed widely throughout France since 1978 are SB0120, SB0134 and SB0121, accounting respectively for 26% and 11% and 6% of all strains. Similar results had been presented in the previous study conducted by Haddad et al [21], only for a shift in SB0134 and SB0121 strain frequency which accounted for 6% and 12% of the total collection until 2000. These 3 main spoligotypes are also present in border countries sharing a long history of trading with France: Italy, where SB0120 strains are the most common and account for 56% of strains [31], and Spain, with SB0121 and SB0134 accounting for 28% and 11% respectively [36].

The similarity of *M bovis* genotypes between countries is probably the consequence of continual sharing of prevalent *M*. *bovis* strains between regions and countries through trading, as would be the case for SB0121 strains, which belong to the Eu2 clonal complex, dominant in the Iberian peninsula, and SB0120 strains which are dominant in Italy [37].

The strain group structure described by combining both genotyping methods delineated 3 main groups of strains with SB0120, SB0134 and SB0121 spoligotypes, with other genetically linked genotypes. The similarity between these strains in the country and neighboring countries can be the marker of long time co-evolution trading action of *M*. *bovis*.

The national prevalence rise of bTB in the last 10 years is mainly explained by the emergence of the disease in a few regions where these SB0120 and SB0134 types seem to spread locally. On the contrary, SB0121 strains are less frequently found in this last period. As a matter of fact, bTB produced by SB0121 types was mainly localized in the North-West during the first period, shifted to the north of France in the second period and to the South and West regions in the third one. Strikingly, SB0121 strains increased in diversity but decreased in number, suggesting less epidemic propagation but a constant spread of this type.

An important result provided by our study is the demonstration of a large genotypic variety within the main spoligotypes. MIRU-VNTR typing confirmed that, as shown before [18,31,38], these main spoligotypes could be split into several MLVA types: 154 in SB0120, 53 in SB0134 and 50 in SB0121.

For SB0120, two main MIRU-VNTR profiles, 5 3 5 3 9 4 5 6 located in Dordogne (South-West) and 5 5 4 3 11 4 5 6 specific of Côte d’Or (Middle-East) came forward, both having emerged in the last 10 years. For SB0134, the current most represented MIRU-VNTR type is 6 4 5 3 6 4 3 6 identified since 2001 principally in Côte d’Or. These 3 MIRU-VNTR profiles account for more than 25% of bTB cases in cattle in France in the last period of the study but they are also present in wildlife-livestock multi-host systems, including wild boar, badgers and cattle.

In this study, we defined the “F4-family” original of Southern of France, which comprises 25 different but closely related spoligotypes, representing 11% of French strains. Some of them have mainly been identified in the last 20 years. SB0821, a member of the “F4-family”, highly concentrated in the South-West, has also been found in other distant regions although it does not seem to only locally spread. Indeed, this spoligotype is largely isolated from cattle of blonde d’Aquitaine breed, original from the Atlantic Pyrenees in the South-West, which in the last 20 years has become an intensively reared suckling cattle breed in France. The movement of animals from cow-calf operator of this breed from the South-West to other regions thus can partly explained the discovery of new SB0821 scattered cases, especially in these last ten years. Some spoligotypes are strictly geographically restricted. This is the case of the “F4-family” spoligotype SB0840, located in Corsica Island, as a consequence of the insular nature of its environment and the very few exchange of animals outwards. Similarly, two other members of the “F4-family”, SB0825 and SB0827, are endemic in Camargue (South), where livestock breeds of fighting bulls are exclusive to this region.

The “F4-family” description indicates the cohabitation of various *M*. *bovis* clonal complexes in different but not too distant French regions. This family, restricted to Southern France, may have diversified in time and nowadays seems to be quite specialized to local ancient breeding practices in this region of the country.

Through this longitudinal study, it is interesting to notice that, on one hand, some genotypes isolated in one period have completely disappeared in the next one and, on the other hand, some genotypes co-exist in the 3 periods. This is the case of SB0134–6 5 5 3 6 4 5 6, present in Ariège-Haute Garonne regions, which seems to spread in the last decade.

In areas where bTB is endemic, we find that the origin of outbreaks is due to a single genotype, i.e. spoligotype and MIRU-VNTR profiles, locally widespread, both in cattle and in wildlife. This tends to prove that the bacterium is not only tailored to a particular animal host, but that in several cases it persists in a multi-host system [22,39]. It is now accepted that the bacterium can evolve in a multi-host system, affecting many different wild animal species such as badgers, wild boar, deer and cattle. As a result, global surveillance and control strategies to fight the disease have to be applied in all susceptible species.

However, besides the spoligotype signatures, additional stable molecular markers such as chromosomal deletions or SNP still need to be described in order to ascribe SB0120, SB0134 or the “F4-family” strains to specific clonal complexes such as African 1 [40], African 2 [41], European 1 [42], and European 2 [43] clonal complexes, defined by a specific SNP and a spoligotype signature.

The molecular identification methods allow for an accurate monitoring of *M*. *bovis* infection progression over time. Although our results provided highly useful knowledge of *M*. *bovis* biodiversity, the applied genotyping methods have some limitations when it comes to explaining inter-herd transmission in high prevalent regions where outbreaks are due to a single genotype. In order to adapt the current control measures at local level, new highly discriminating SNP typing methods based on complete genome sequencing of bacteria should be considered as an alternative [44]. In term, as depicted by Kohl and colleagues for *M*. *tuberculosis* [45], WGS through adapted data standardization for *M*. *bovis*, will eventually replace traditional molecular typing facilitating high-resolution discrimination.

The wide genotypic variety within spoligotype groups and the strong genotype regionalization made molecular typing an excellent means for tracing up and for easily explaining outbreak origin (relapse, movement, inter-species transmission) in France. But most importantly, this strain characterization added new knowledge of the current *M*. *bovis* agents inducing the recent residual bTB in France, which is at present spreading in multi-host systems in highly productive cattle industry regions.

To understand the persistence and regionalization of certain strains which were capable of crossing the species barrier and infect wildlife, works based on the complete genome sequencing of *M*. *bovis* strains are underway. Mutations in the genome of the bacteria on *loci* described as having a link with the modulation of virulence in *M*. *tuberculosis* and *M*. *bovis* could allow the testing of the hypothesis that the persistence of bTB may be associated to a genetic change leading to a higher virulence [46]. Indeed, recent studies conducted in Northern Ireland have shown that the patterns of lesion could vary depending on the genotype of the infecting *M*. *bovis*, some of them appear to have increased virulence traits [47]. To validate the possibility of virulence genotypes deducted *in silico*, the study of their phenotypes could be performed using different models such as macrophage infection or infection *in vivo* models in mice and in cattle.

In conclusion, together with conventional epidemiology, the molecular data obtained until now greatly supports and gives clues for ameliorating the national eradication campaign and tackle the disease by focusing on emergent traits of French modern tuberculosis.

## Supporting Information

S1 Dataset Sheet 1. Most common French spoligotypes. This table presents the number, percentage and profiles of spoligotypes identified more than 30 times in France during the 1978–2013 period. * represents spoligotypes associated with “F4-family”. ^a^ refer to all the strains less represented in France. Sheet 2. Others. This table presents the spoligotype profiles of strains identified less than 30 times in France during the1978–2013 period. * represents spoligotypes associated with F4-family.
(XLSX)

S1 Fig Minimum spanning tree (MST) of the 778 *M*. *bovis* SB0120 strains isolated and typed between 1978 and 2013 (A) and Geographical distribution of 1230 SB0120 strains outbreaks in France (B). A: 154 genotype profiles obtained by combination of spoligotyping and VNTR typing. The colored nodes identify the principal VNTR types (represented more than 5 times). The size of the nodes is proportional to the number of strains identified with the MLVA profile. Distances between nodes represent distances between profiles. B: The shade of red reflects the percentage of SB0120 strains isolated in each “department” compared to the totality of SB0120 isolated in the entire territory for the given period of time. The first map represents the 117 *M*. *bovis* strains isolated in France from 1978 to 1990. The second map represents the 342 *M*. *bovis* strains isolated in France from 1991 to 2000. The third map represents the 771 *M*. *bovis* strains in France from 2001 to 2013. Circles on the maps indicate the localization of the principal VNTR types.
(TIF)

S2 Fig Minimum spanning tree (MST) of the 270 *M*. *bovis* SB0134 strains isolated and typed between 1978 and 2013 (A) and Geographical distribution of 533 SB0134 strains outbreaks in France (B). A: 53 genotype profiles obtained by combination of spoligotyping and VNTR typing. The colored nodes identify the principal VNTR types (represented more than 2 times). The size of the nodes is proportional to the number of strains identified with the MLVA profile. Distances between nodes represent distances between profiles. B: The shade of red reflects the percentage of SB0134 strains isolated in each “department” compared to the totality of SB0134 isolated in the entire territory for the given period of time. The first map represents the 94 *M*. *bovis* strains isolated in France from 1978 to 1990. The second map represents the 92 *M*. *bovis* strains isolated in France from 1991 to 2000. The third map represents the 347 *M*. *bovis* strains in France from 2001 to 2013. Circles on the maps indicate the localization of the principal VNTR types.
(TIF)

S3 Fig Minimum spanning tree (MST) of the 154 *M*. *bovis* SB0121 strains isolated and typed between 1978 and 2013 (A) and Geographical distribution of 302 SB0121 strains outbreaks in France (B). **A:** 50 genotype profiles obtained by combination of spoligotyping and VNTR typing. The colored nodes identify the principal VNTR types (represented more than 2 times). The size of the nodes is proportional to the number of strains identified with the MLVA profile. Distances between nodes represent distances between profiles. B. The shade of red reflects the percentage of SB0121 strains isolated in each “department” compared to the totality of SB0121 isolated in the entire territory for the given period of time. The first map represents the 54 *M*. *bovis* strains isolated in France from 1978 to 1990. The second map represents the 140 *M*. *bovis* strains isolated in France from 1991 to 2000. The third map represents the 108 *M*. *bovis* strains in France from 2001 to 2013. Circles on the maps indicate the localization of the principal VNTR types.
(TIF)

S4 Fig Minimum spanning tree (MST) of the 530 *M*. *bovis* strains which belong to the “F4-family” isolated between 1978 and 2013 (A) and Geographical distribution of the “F4-family” strains outbreaks in France (B). A: 137 genotype profiles obtained by combination of spoligotyping and VNTR typing (25 spoligotypes and 106 MLVA profiles). The colored nodes identify the 12 principal spoligotypes (represented more than 6 times). The size of the nodes is proportional to the number of strains identified with the genotype. Distances between nodes represent distances between profiles. B: The shade of red reflects the percentage of “F4-family” strains isolated in each “department” compared to the totality of “F4-family” isolated in the entire territory for the given period of time. The first map represents the 12 “F4-family” strains isolated in France from 1978 to 1990. The second map represents the 150 “F4-family” strains isolated in France from 1991 to 2000. The third map represents the 368 “F4-family” strains in France from 2001 to 2013. Circles on the maps indicate the localization of the spoligotypes.
(TIF)
