# Supplementary figures and images for: Genetic Evolution of Mycobacterium bovis Causing Tuberculosis in Livestock and Wildlife in France since 1978
Source: PLoS One. 2015 Feb 6;10(2):e0117103. doi: 10.1371/journal.pone.0117103 (PMC4319773; doi:10.1371/journal.pone.0117103)

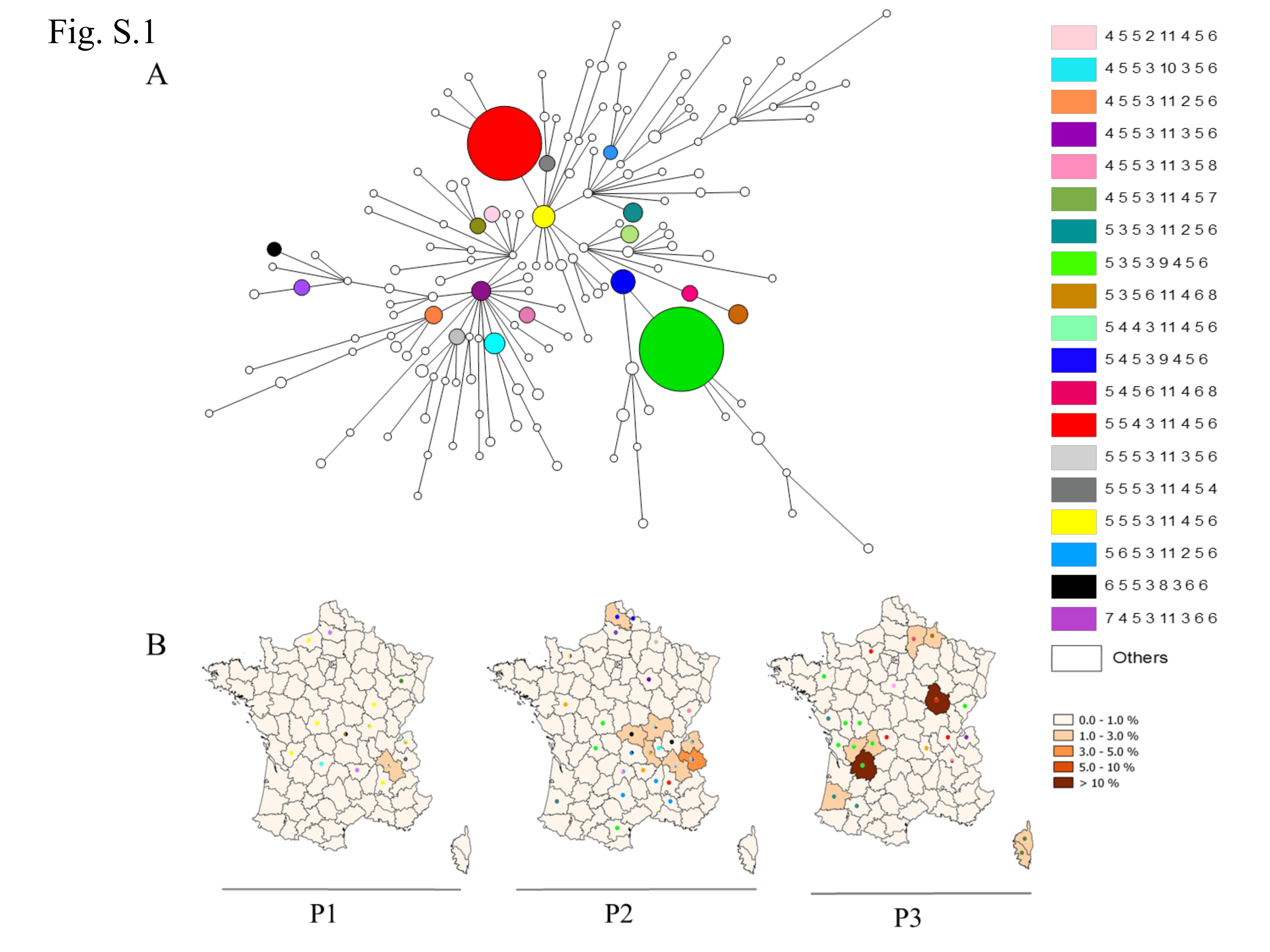

Supplement: S1 Fig — A: 154 genotype profiles obtained by combination of spoligotyping and VNTR typing. The colored nodes identify the principal VNTR types (represented more than 5 times). The size of the nodes is proportional to the number of strains identified with the MLVA profile. Distances between nodes represent distances between profiles. B: The shade of red reflects the percentage of SB0120 strains isolated in each “department” compared to the totality of SB0120 isolated in the entire territory for the given period of time. The first map represents the 117 M. bovis strains isolated in France from 1978 to 1990. The second map represents the 342 M. bovis strains isolated in France from 1991 to 2000. The third map represents the 771 M. bovis strains in France from 2001 to 2013. Circles on the maps indicate the localization of the principal VNTR types. (TIF) [file pone.0117103.s002.tif]

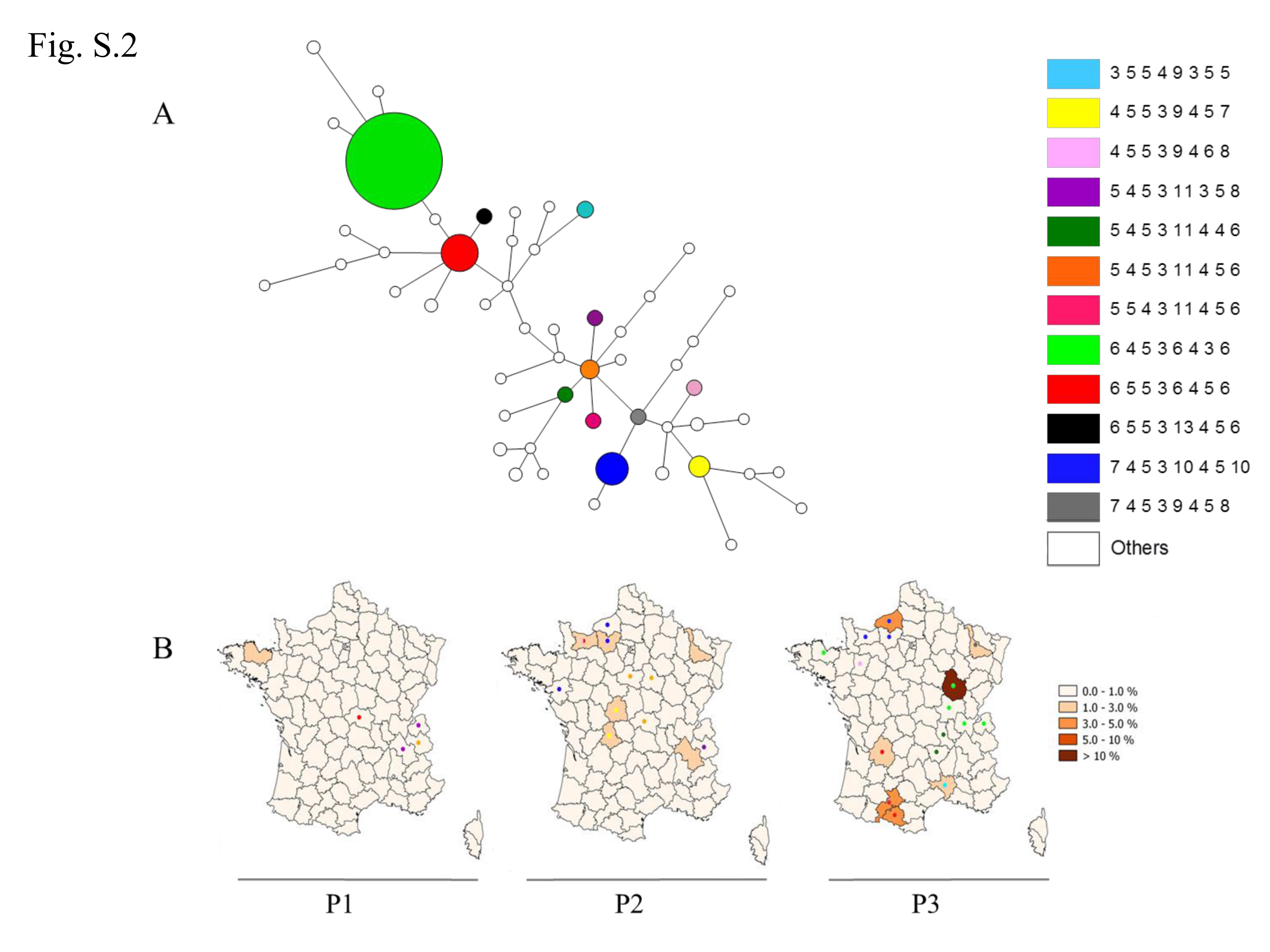

Supplement: S2 Fig — A: 53 genotype profiles obtained by combination of spoligotyping and VNTR typing. The colored nodes identify the principal VNTR types (represented more than 2 times). The size of the nodes is proportional to the number of strains identified with the MLVA profile. Distances between nodes represent distances between profiles. B: The shade of red reflects the percentage of SB0134 strains isolated in each “department” compared to the totality of SB0134 isolated in the entire territory for the given period of time. The first map represents the 94 M. bovis strains isolated in France from 1978 to 1990. The second map represents the 92 M. bovis strains isolated in France from 1991 to 2000. The third map represents the 347 M. bovis strains in France from 2001 to 2013. Circles on the maps indicate the localization of the principal VNTR types. (TIF) [file pone.0117103.s003.tif]

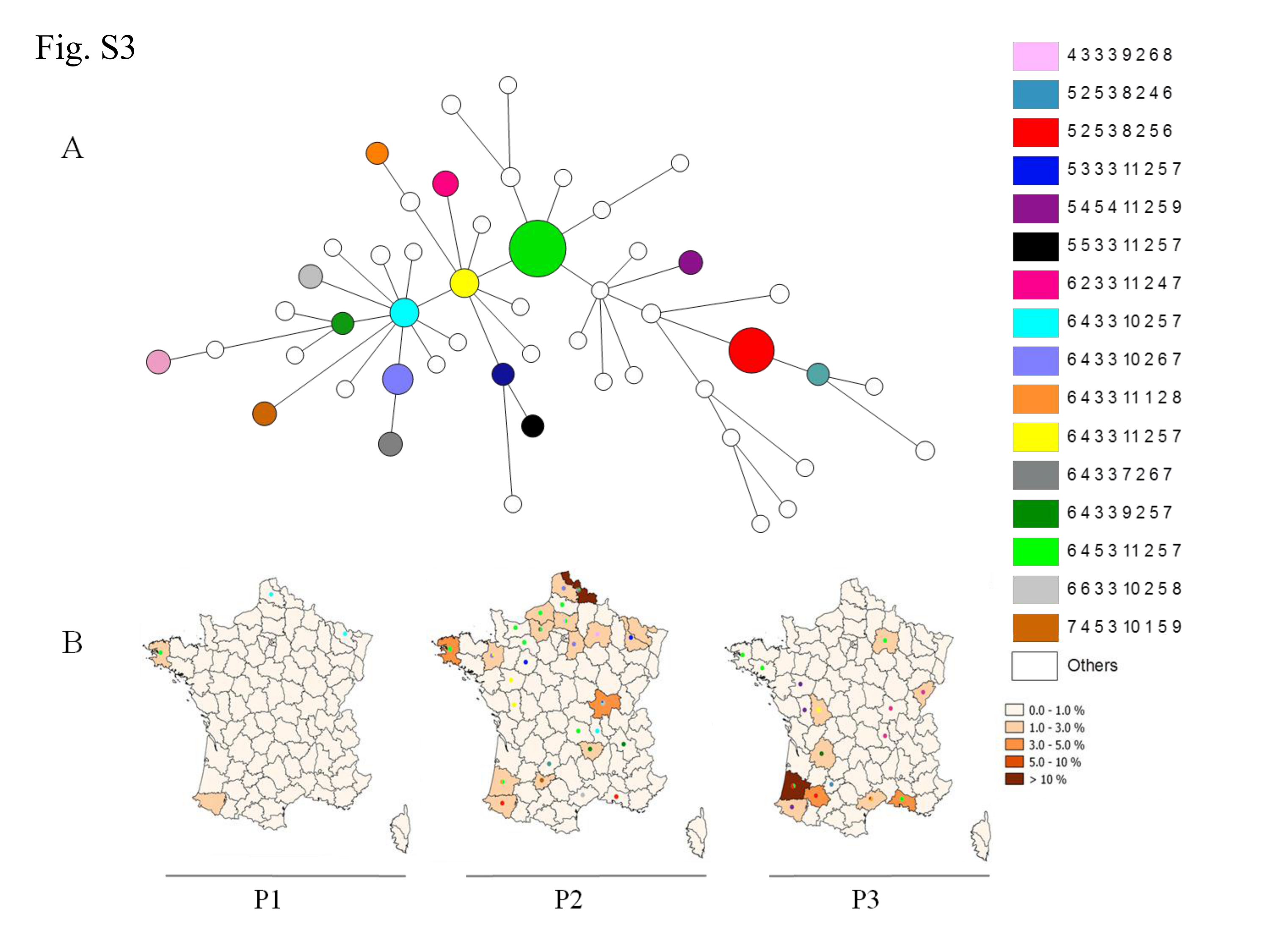

Supplement: S3 Fig — A: 50 genotype profiles obtained by combination of spoligotyping and VNTR typing. The colored nodes identify the principal VNTR types (represented more than 2 times). The size of the nodes is proportional to the number of strains identified with the MLVA profile. Distances between nodes represent distances between profiles. B. The shade of red reflects the percentage of SB0121 strains isolated in each “department” compared to the totality of SB0121 isolated in the entire territory for the given period of time. The first map represents the 54 M. bovis strains isolated in France from 1978 to 1990. The second map represents the 140 M. bovis strains isolated in France from 1991 to 2000. The third map represents the 108 M. bovis strains in France from 2001 to 2013. Circles on the maps indicate the localization of the principal VNTR types. (TIF) [file pone.0117103.s004.tif]

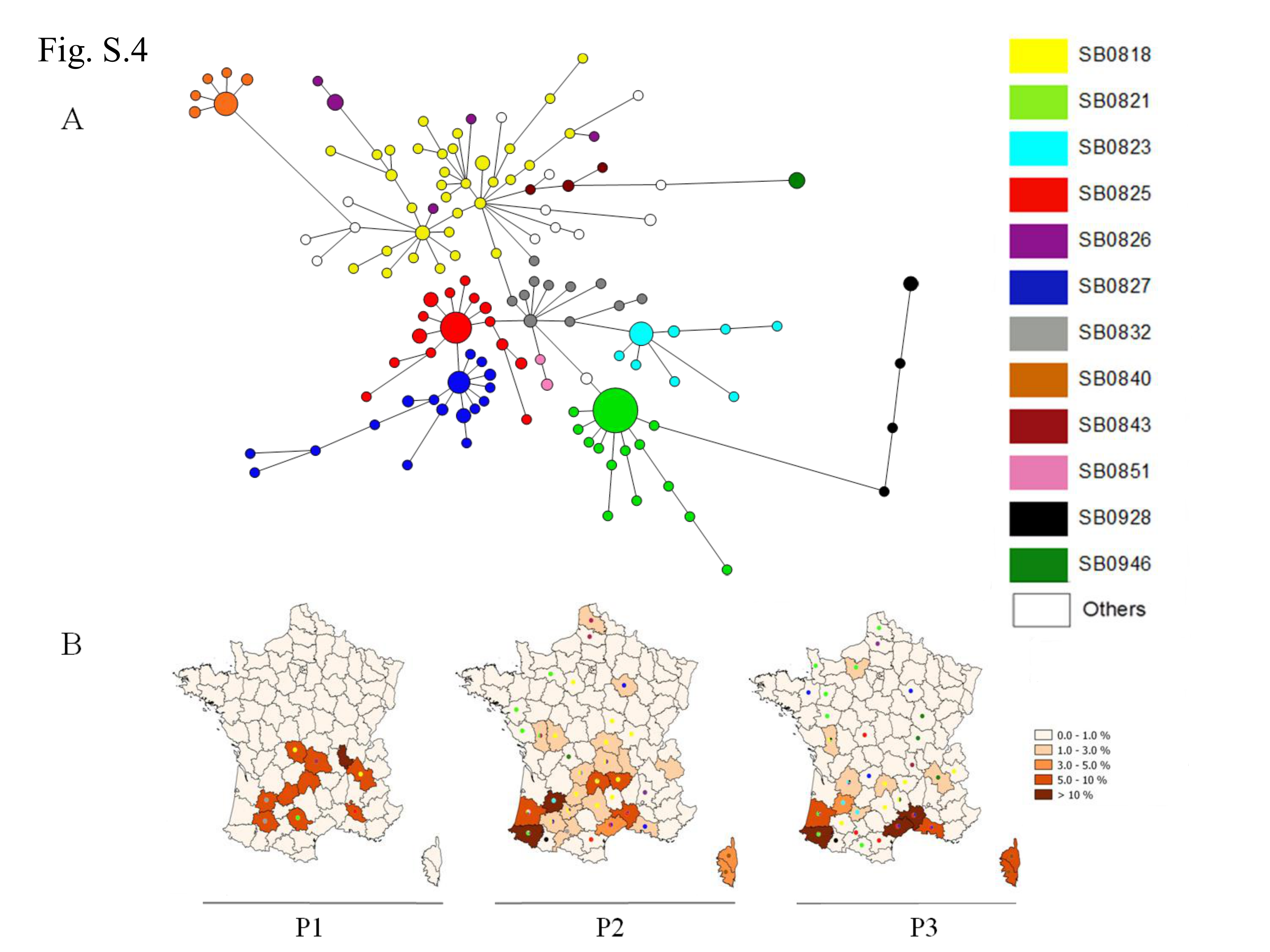

Supplement: S4 Fig — A: 137 genotype profiles obtained by combination of spoligotyping and VNTR typing (25 spoligotypes and 106 MLVA profiles). The colored nodes identify the 12 principal spoligotypes (represented more than 6 times). The size of the nodes is proportional to the number of strains identified with the genotype. Distances between nodes represent distances between profiles. B: The shade of red reflects the percentage of “F4-family” strains isolated in each “department” compared to the totality of “F4-family” isolated in the entire territory for the given period of time. The first map represents the 12 “F4-family” strains isolated in France from 1978 to 1990. The second map represents the 150 “F4-family” strains isolated in France from 1991 to 2000. The third map represents the 368 “F4-family” strains in France from 2001 to 2013. Circles on the maps indicate the localization of the spoligotypes. (TIF) [file pone.0117103.s005.tif]
